# Supplementary material for: Smooth Interpolating Curves with Local Control and Monotone Alternating Curvature
Source: Comput Graph Forum. 2022 Oct 6;41(5):25–38. doi: 10.1111/cgf.14600 (PMC9827861; doi:10.1111/cgf.14600)
Supplement: Supplementary file 1 — Supplement Material [file CGF-41-25-s001.zip › Local-Smooth-Interpolating-MonoCurvature/extern/clothoids/docs/api-cpp/unabridged_api.html]

Full API — Clothoids v2.0.9

### Navigation

- index
- toc
- Clothoids »
- Full API

# Full API¶

## Namespaces¶

- Namespace G2lib
  - Detailed Description
  - Classes
  - Enums
  - Functions
  - Typedefs
  - Variables

## Classes and Structs¶

- Class AABBtree
  - Class Documentation

- Class AsyPlot
  - Class Documentation

- Class BaseCurve
  - Inheritance Relationships
    - Derived Types
  - Class Documentation

- Class BBox
  - Class Documentation

- Class Biarc
  - Inheritance Relationships
    - Base Type
  - Class Documentation

- Class BiarcList
  - Inheritance Relationships
    - Base Type
  - Class Documentation

- Class CircleArc
  - Inheritance Relationships
    - Base Type
  - Class Documentation

- Class ClothoidCurve
  - Inheritance Relationships
    - Base Type
  - Class Documentation

- Class ClothoidList
  - Inheritance Relationships
    - Base Type
  - Class Documentation

- Class ClothoidSplineG2
  - Class Documentation

- Class G2solve2arc
  - Class Documentation

- Class G2solve3arc
  - Class Documentation

- Class G2solveCLC
  - Class Documentation

- Class LineSegment
  - Inheritance Relationships
    - Base Type
  - Class Documentation

- Class PolyLine
  - Inheritance Relationships
    - Base Type
  - Class Documentation

- Class Solve2x2
  - Class Documentation

- Class Triangle2D
  - Class Documentation

## Enums¶

- Enum CurveType
  - Enum Documentation

## Functions¶

- Function G2lib::Atanc
  - Function Documentation

- Function G2lib::Atanc\_D
  - Function Documentation

- Function G2lib::Atanc\_DD
  - Function Documentation

- Function G2lib::Atanc\_DDD
  - Function Documentation

- Function G2lib::build\_guess\_theta
  - Function Documentation

- Function G2lib::collision
  - Function Documentation

- Function G2lib::collision\_ISO
  - Function Documentation

- Function G2lib::collision\_SAE
  - Function Documentation

- Function G2lib::Cosc
  - Function Documentation

- Function G2lib::Cosc\_D
  - Function Documentation

- Function G2lib::Cosc\_DD
  - Function Documentation

- Function G2lib::Cosc\_DDD
  - Function Documentation

- Function G2lib::FresnelCS(real\_type, real\_type&, real\_type&)
  - Function Documentation

- Function G2lib::FresnelCS(int\_type, real\_type, real\_type \*, real\_type \*)
  - Function Documentation

- Function G2lib::GeneralizedFresnelCS(int\_type, real\_type, real\_type, real\_type, real\_type \*, real\_type \*)
  - Function Documentation

- Function G2lib::GeneralizedFresnelCS(real\_type, real\_type, real\_type, real\_type&, real\_type&)
  - Function Documentation

- Function G2lib::intersect
  - Function Documentation

- Function G2lib::intersect\_ISO
  - Function Documentation

- Function G2lib::intersect\_SAE
  - Function Documentation

- Function G2lib::intersectCircleCircle
  - Function Documentation

- Function G2lib::isCounterClockwise
  - Function Documentation

- Function G2lib::isPointInTriangle
  - Function Documentation

- Function G2lib::minmax3
  - Function Documentation

- Function G2lib::noAABBtree
  - Function Documentation

- Function G2lib::operator<<(ostream\_type&, Biarc const&)
  - Function Documentation

- Function G2lib::operator<<(ostream\_type&, BiarcList const&)
  - Function Documentation

- Function G2lib::operator<<(ostream\_type&, CircleArc const&)
  - Function Documentation

- Function G2lib::operator<<(ostream\_type&, ClothoidCurve const&)
  - Function Documentation

- Function G2lib::operator<<(ostream\_type&, ClothoidSplineG2 const&)
  - Function Documentation

- Function G2lib::operator<<(ostream\_type&, ClothoidList const&)
  - Function Documentation

- Function G2lib::operator<<(ostream\_type&, BBox const&)
  - Function Documentation

- Function G2lib::operator<<(ostream\_type&, LineSegment const&)
  - Function Documentation

- Function G2lib::operator<<(ostream\_type&, PolyLine const&)
  - Function Documentation

- Function G2lib::operator<<(ostream\_type&, Triangle2D const&)
  - Function Documentation

- Function G2lib::pointInsideCircle
  - Function Documentation

- Function G2lib::projectPointOnCircle
  - Function Documentation

- Function G2lib::projectPointOnCircleArc
  - Function Documentation

- Function G2lib::rangeSymm
  - Function Documentation

- Function G2lib::Sinc
  - Function Documentation

- Function G2lib::Sinc\_D
  - Function Documentation

- Function G2lib::Sinc\_DD
  - Function Documentation

- Function G2lib::Sinc\_DDD
  - Function Documentation

- Function G2lib::solveLinearQuadratic
  - Function Documentation

- Function G2lib::solveLinearQuadratic2
  - Function Documentation

- Function G2lib::xy\_to\_guess\_angle
  - Function Documentation

- Function G2lib::yesAABBtree
  - Function Documentation

## Variables¶

- Variable G2lib::CurveType\_name
  - Variable Documentation

- Variable G2lib::intersect\_with\_AABBtree
  - Variable Documentation

- Variable G2lib::m\_1\_sqrt\_pi
  - Variable Documentation

- Variable G2lib::machepsi
  - Variable Documentation

- Variable G2lib::machepsi10
  - Variable Documentation

- Variable G2lib::machepsi100
  - Variable Documentation

- Variable G2lib::machepsi1000
  - Variable Documentation

- Variable G2lib::sqrtMachepsi
  - Variable Documentation

## Defines¶

- Define CLOTHOIDS\_dot\_HH
  - Define Documentation

- Define G2LIB\_DEBUG\_MESSAGE
  - Define Documentation

- Define G2LIB\_USE\_CXX11
  - Define Documentation

- Define GLIB2\_TOL\_ANGLE
  - Define Documentation

## Typedefs¶

- Typedef G2lib::int\_type
  - Typedef Documentation

- Typedef G2lib::IntersectList
  - Typedef Documentation

- Typedef G2lib::Ipair
  - Typedef Documentation

- Typedef G2lib::istream\_type
  - Typedef Documentation

- Typedef G2lib::ostream\_type
  - Typedef Documentation

- Typedef G2lib::real\_type
  - Typedef Documentation

### Quick search

### Table of Contents

- Matlab Interface Manual
- C++ API
- MATLAB API

«
hide menu

menu
sidebar
»

### Navigation

- index
- toc
- Clothoids »
- Full API

© Copyright 2021, Enrico Bertolazzi and Marco Frego.
Created using Sphinx 4.2.0.
